# Supplementary material for: Forecasting COVID-19 activity in Australia to support pandemic response: May to October 2020
Source: Sci Rep. 2023 May 30;13:8763. doi: 10.1038/s41598-023-35668-6 (PMC10228456; doi:10.1038/s41598-023-35668-6)
Supplement: Supplementary file 1 — Supplementary Information. [file 41598_2023_35668_MOESM1_ESM.pdf]

S Supplementary figures and results

S.1 Forecast sharpness

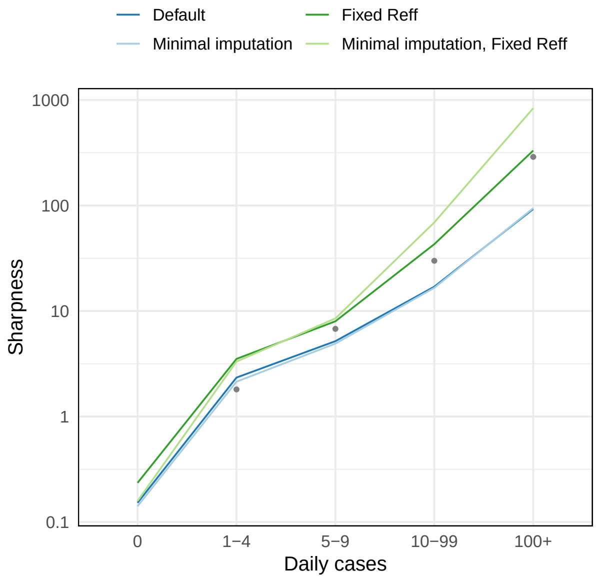

Figure S1: Forecast sharpness binned by the number of cases ultimately recorded for the day of the prediction. Mean recorded case numbers for each (non-zero) bin are shown as grey points.

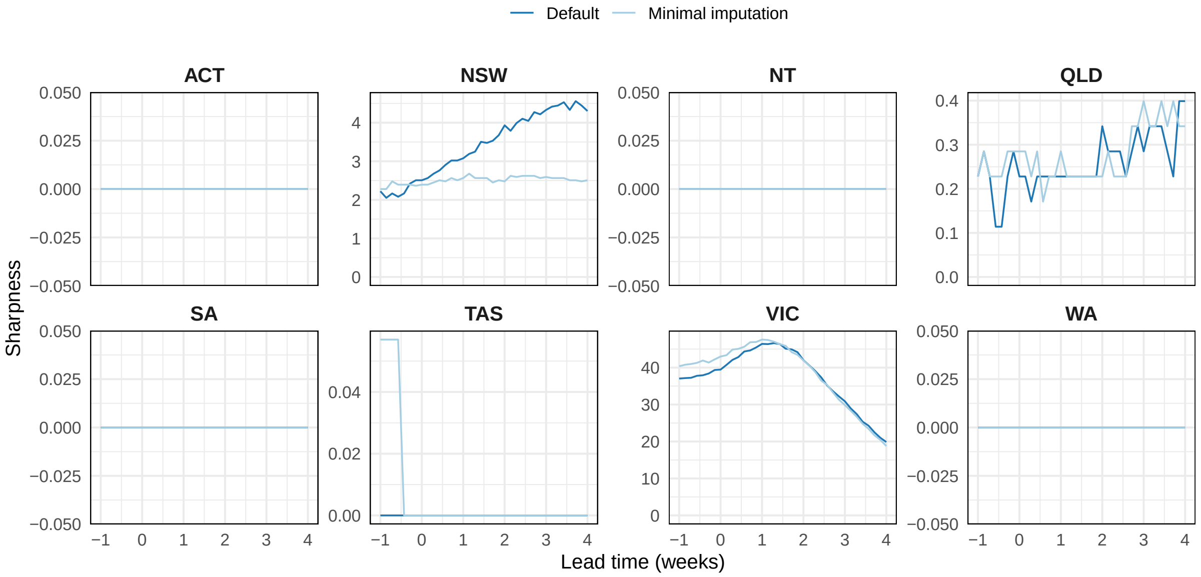

Figure S2: Forecast sharpness by lead time for the default and minimal imputation models, where the R_{eff} distribution gradually trends back towards the transmission potential (TP). During the second wave in Victoria, strong public health measures and social restrictions markedly reduced the TP, which caused forecasts to become sharper at longer lead times.

As a general rule, forecasts usually grow more uncertain as they predict further ahead [1]. However, the forecasts presented here for longer lead times tended to exhibit little growth in uncertainty, or even grow more certain (Figure S2).

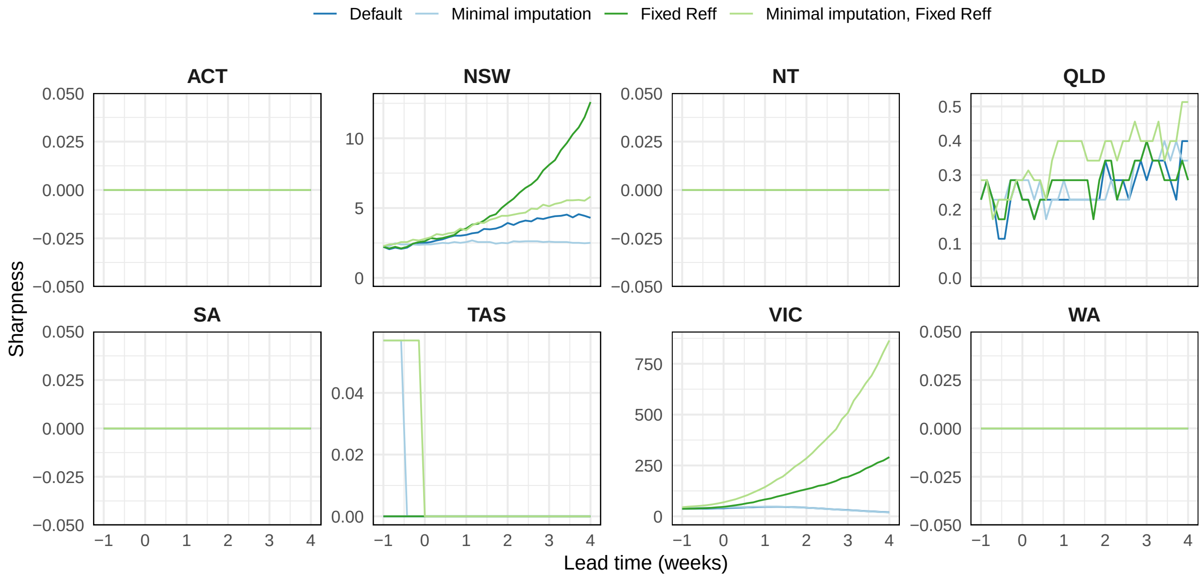

Figure S3: Forecast sharpness by lead time, shown separately for each forecast model.

For jurisdictions that primarily reported zero cases on a given day, the absence of epidemic growth meant that the forecast trajectories tended to local extinction and did not allow for a subsequent rebound. Accordingly, forecast sharpness in these jurisdictions remained very close to zero (i.e., no uncertainty) for all lead times.

For jurisdictions that reported substantial COVID-19 activity, forecasts for longer lead times either (a) exhibited little increase in uncertainty (New South Wales, Queensland); or (b) grew more certain but did not approach zero (Victoria). These jurisdictions imposed intense mobility and gathering restrictions in response to local COVID-19 activity, and the reduced contact rates in the general population meant that the model reproduction number tended to decrease below unity over the forecast horizon.

The forecasts did, however, grow more uncertain as the daily number of cases increased (Figure S1). And forecasts generated under the assumption that transmission would be sustained at the current active-case R_{eff} grew more uncertain for longer lead times (Figure S3, green lines).

S.2 Forecast bias

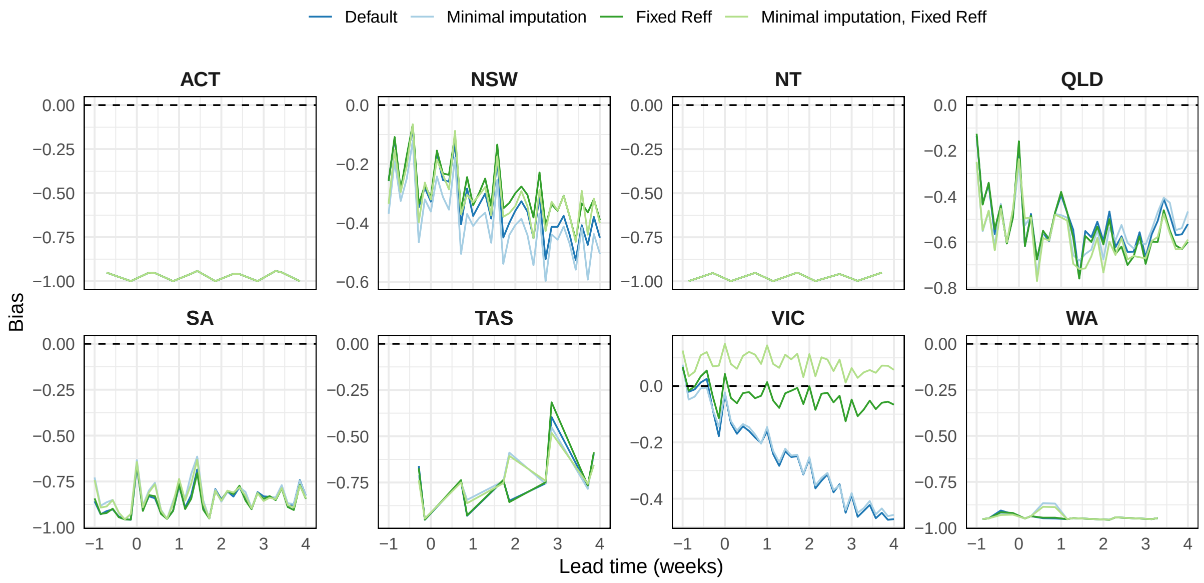

Figure S4: Forecast bias by lead time, for days where at least one case was reported.

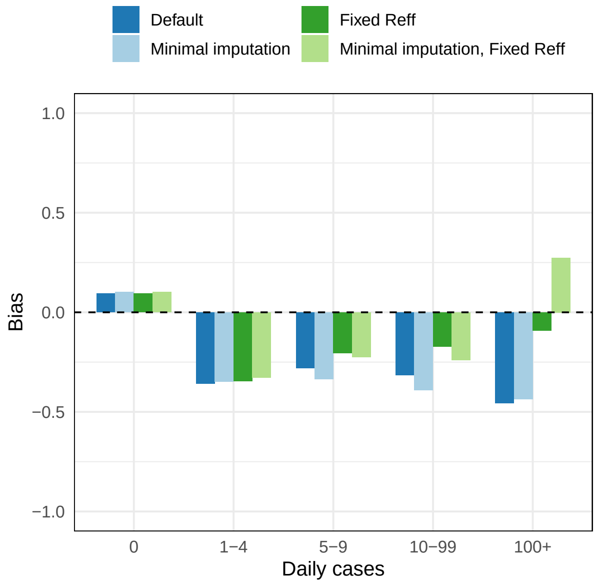

Figure S5: Forecast bias binned by the number of cases ultimately recorded for the day of the prediction.

Bias was defined so that 0 indicated an unbiased forecast, whose probability mass was distributed equally above and below the data, −1 indicated a forecast that completely under-estimated the data, and 1 indicated a forecast that completely over-estimated the data.

For jurisdictions that primarily reported zero cases on a given day (e.g., Australian Capital Territory, Northern Territory, South Australia, Tasmania, Western Australia) the forecast bias for days where one or more cases were reported is close to negative one (Figure S4). This indicates a deliberate model feature: our careful separation of the *potential* for reintroduction from the *consequence* of reintroduction (Section 4.3). For

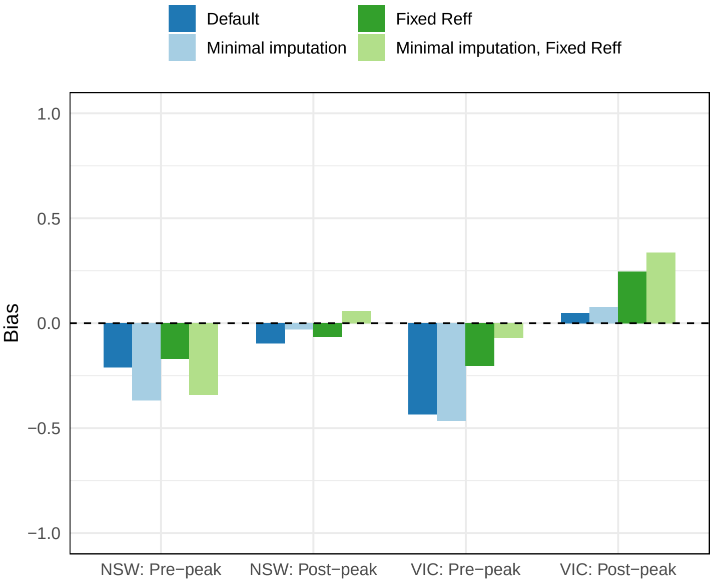

Figure S6: Forecast bias for the second wave in New South Wales and Victoria, reported seperately for data dates before and after the observed peak in each state (NSW: 29 July; VIC: 27 July).

jurisdictions which had achieved local elimination and for which there was no evidence of reintroduction, the forecasts did not allow for subsequent epidemic activity.

In jurisdictions that saw substantial COVID-19 activity (New South Wales, Queensland, Victoria) the forecast bias was also negative and grew in magnitude as the lead time increased, but did not approach negative one (Figure S4). Forecasts exhibited a small positive bias on days where zero cases were reported (Figure S5, left-most category), because the forecasts always included some uncertainty and did not allow negative case counts. As the number of daily cases increased, the forecast bias became negative and grew in magnitude (Figure S5). When we consider the pre-peak and post-peak intervals of the second wave in Victoria and in New South Wales — the only period where any jurisdiction reported more than 100 local cases in a single day — we find that the forecast bias was negative prior to the peak and was approximately zero after the peak (Figure S6 and Figure S7). This suggests that the negative bias was due to a combination of factors.

During the second wave, the active-case R_{eff} was consistently higher than the whole-population transmission potential. So while the restrictions imposed during the second wave did reduce the active-case R_{eff} over time, and eventually brought the second wave to an end, our model over-estimated the decrease in local transmission over the forecast horizon. Despite this negative bias, the model consistently out-performed forecasts generated under the assumption that local transmission would be sustained at the current active-case R_{eff} (see Section S.3 for details).

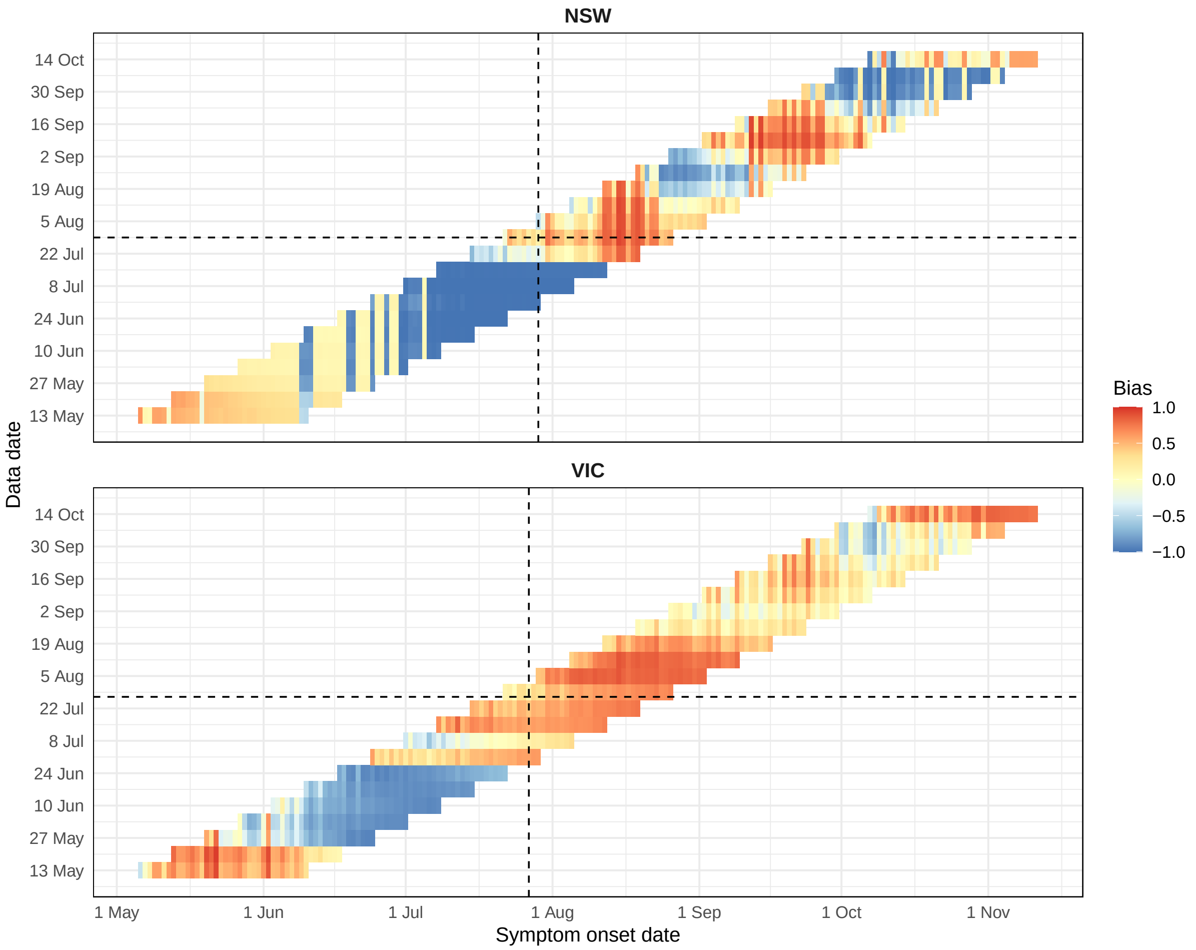

Figure S7: Forecast bias for the second wave in New South Wales and Victoria, shown separately for each day in the forecast horizon (x-axis) for each data date (y-axis). Dashed lines indicate the timing of the peak in each jurisdiction (NSW: 29 July; VIC: 27 July). There is a general trend to underestimate case counts (negative bias, blue) prior to the peak and to overestimate case counts (positive bias, red) after the peak. However, forecasts made in the few weeks prior to the observed peaks also exhibit positive bias.

S.3 CRPS skill scores

S.3.1 Forecast skill by reported daily cases

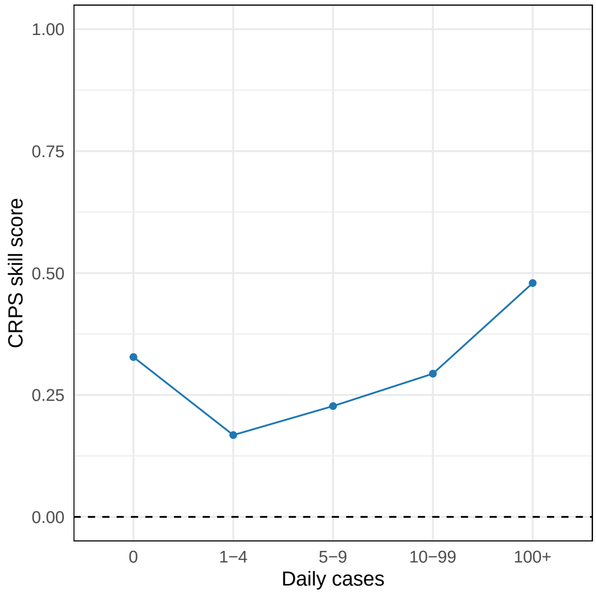

Figure S8: CRPS skill scores binned by the number of cases ultimately recorded for the day of the prediction.

We considered forecast skill as a function of the number of cases ultimately recorded on each day in each jurisdiction (comprising 1,688 observations). We divided the daily case numbers into five bins: 0 cases (n = 1298, 76.9%); 1–4 cases (n = 186, 11.0%); 5–9 cases (n = 57, 3.4%); 10–99 cases (n = 91, 5.4%); and 100+ cases (n = 56, 3.3%). The forecasts always out-performed the historical benchmark. The forecast skill across all jurisdictions was highest when daily case numbers were zero or ≥ 10, and was lower for days where 1–9 cases were reported (Figure S8). By using our model to predict how transmission would change over the forecast horizon (relative to the current active-case R_{eff} estimate) forecast performance was greatly increased (Figure S9, compare blue lines to green lines).

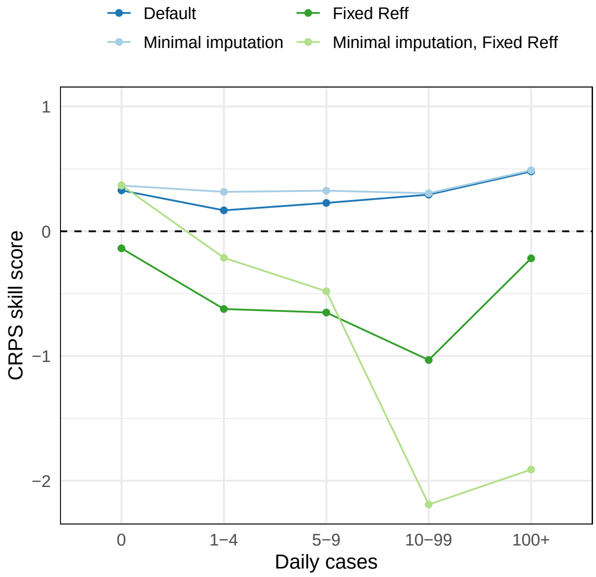

Figure S9: CRPS skill scores binned by the number of cases ultimately recorded for the day of the prediction, shown separately for each forecast model. The default model is slightly out-performed by the minimal imputation model when there are 1–9 cases per day, but has near-identical performance for higher case numbers and when there are zero cases.

S.3.2 Forecast skill when conditioning on incomplete data

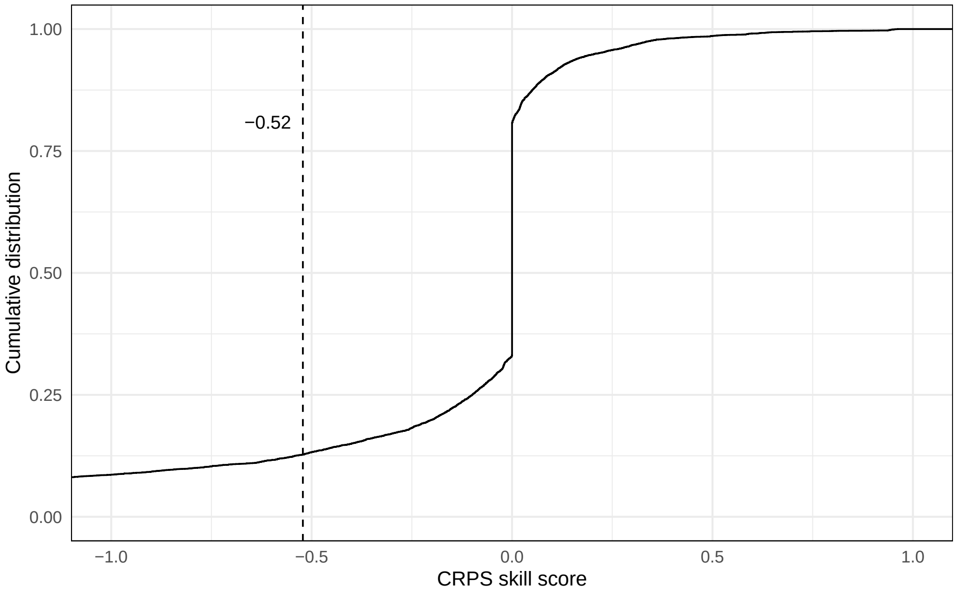

Figure S10: The cumulative distribution of CRPS skill scores for the minimal imputation model (which was not conditioned on data for dates where the detection probability was $< 95\%$) relative to the default model. The mean skill score was -0.52 (dashed vertical line).

We estimated the delay distribution from symptom onset to case notifications being reported, and imputed the symptom onset data for cases where this field was not yet completed (see Section 4.1). The forecasting model was then conditioned on case counts, after accounting for under-reporting, for those dates where at least 50% of cases were estimated to have been reported (see Section 4.2). To determine how this treatment of the case data affected forecast skill, we used a separate "minimal imputation" model that was only conditioned on case counts for dates where at least 95% of cases were estimated to have been reported. We calculated CRPS skill scores for the "minimal imputation" model, relative to the original model, for each of the 7,488 predictions (8 jurisdictions, 26 data dates, 36-day forecasting window). The cumulative distribution of these skill scores is shown in Figure S10.

The minimal imputation model out-performed the default model for 19.3% of the predictions (median skill score of 0.09), yielded identical performance for 47.6% of the predictions, and was out-performed by the default model for 33.2% of the predictions (median skill score of -0.32). Identical performance primarily occurred on days with zero cases (98.1%), with the remainder occurring on days with 1–3 cases. The mean skill score was -0.52. This indicates that imputing symptom onset dates and accounting for delayed ascertainment provided additional information to that available in raw time-series data.

There were 25 predictions where the skill score was $\geq 90\%$, 23 of which pertained to a single data date for NSW (17 June) or Queensland (6 May).

For New South Wales, the default model was conditioned on data for several days where 1 local case was reported, while the minimal imputation model was not conditioned on these data (because fewer than 95% of cases were estimated to have been reported for these dates). Accordingly, the default model predicted a subsequent increase in local cases, while the minimal imputation model predicted no local cases (95% CrI: 0–1 cases).

These data were revised in subsequent data extracts, and each of the days with 1 reported local case now had no local cases.

The same explanation applies to Queensland, where only 2 cases were reported over the forecast horizon. The default model was conditioned on data for several days where 1 local case was reported, and predicted a larger increase in cases than the minimal imputation model, which was not conditioned on these data. Both sets of forecasts had identical medians (0 cases) and 50% CrIs (0–0 cases), but the default model had wider 95% CrIs (0–18 cases) than the minimal imputation model (0–4 cases).

S.3.3 Overall forecast skill in each jurisdiction

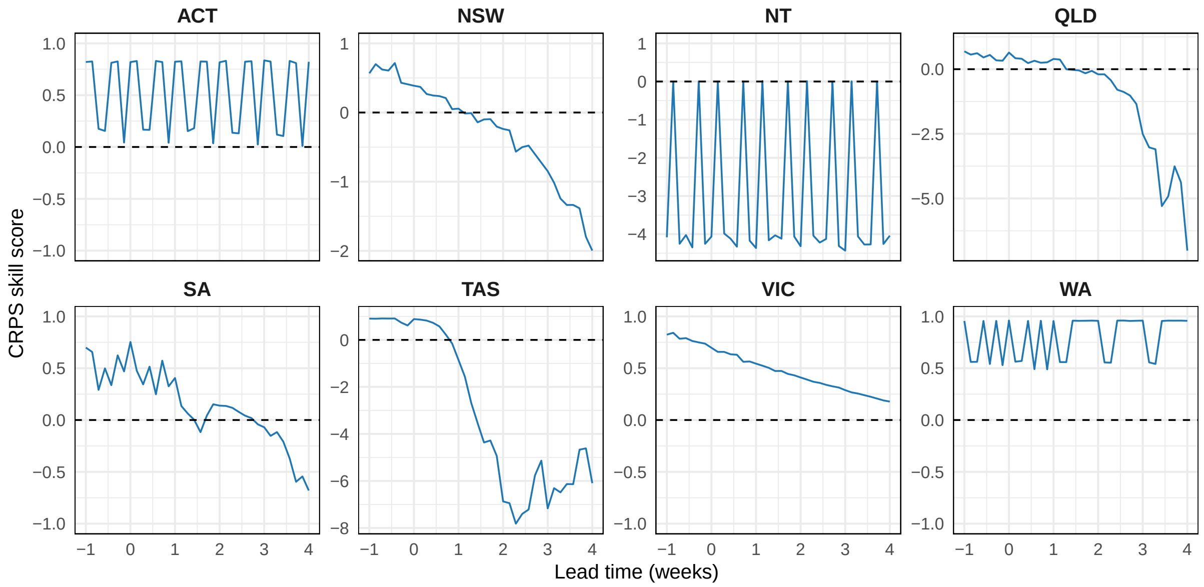

Figure S11: CRPS skill scores by forecast lead time, shown separately for each jurisdiction.

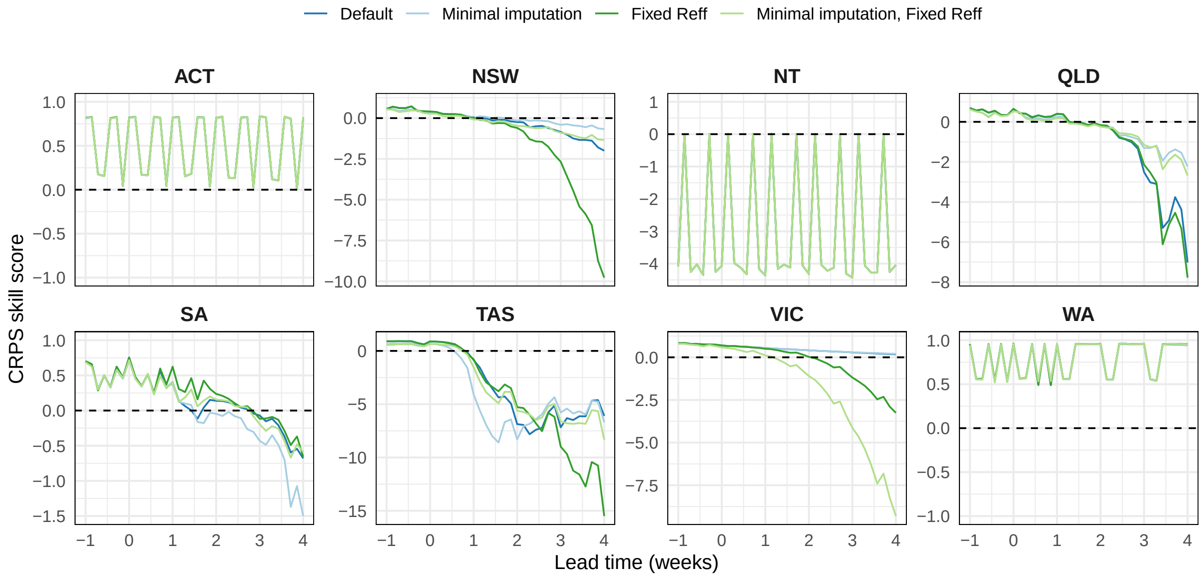

Figure S12: CRPS skill scores by forecast lead time, shown for each forecast model. Allowing the R_{eff} distribution to trend back towards the population mean (blue) consistently out-performed keeping the R_{eff} distribution fixed from the forecasting date onward (green). Conditioning on, and not conditioning on, the most recent case counts (dark shades and light shades, respectively) did not exhibit a consistent trend across jurisdictions.

We measured forecast skill relative to an historical benchmark forecast (see Section 4.4). Of the jurisdictions that primarily reported zero cases on a given day, the forecasts consistently out-performed the historical benchmark for all lead times in the Australian Capital Territory and Western Australia, and out-performed the historical benchmark for 1-week lead times in South Australia and Tasmania (Figure S11). The Northern Territory reported only three cases in the first wave and three cases in the study period

(linked to separate importation events); these were ideal circumstances for the historical benchmark, which out-performed the model forecasts. For Tasmania, which experienced a local outbreak in the first wave and reported only two cases in the study period, the forecasts exhibited improved performance for lead times of up to one week, and exhibited decreased performance for longer lead times.

Of the three jurisdictions that experienced substantial COVID-19 activity (New South Wales, Queensland, Victoria), the forecasts exhibited improved performance for shorter lead times (Figure S11). The forecasts for Victoria, which experienced the largest local outbreak, substantially out-performed the historical benchmark at even the longest lead times. For New South Wales and Victoria, the model consistently out-performed forecasts generated under the assumption that local transmission would be sustained at the current active-case R_{eff} (Figure S12).

S.3.4 Forecast skill before and after the second wave peak

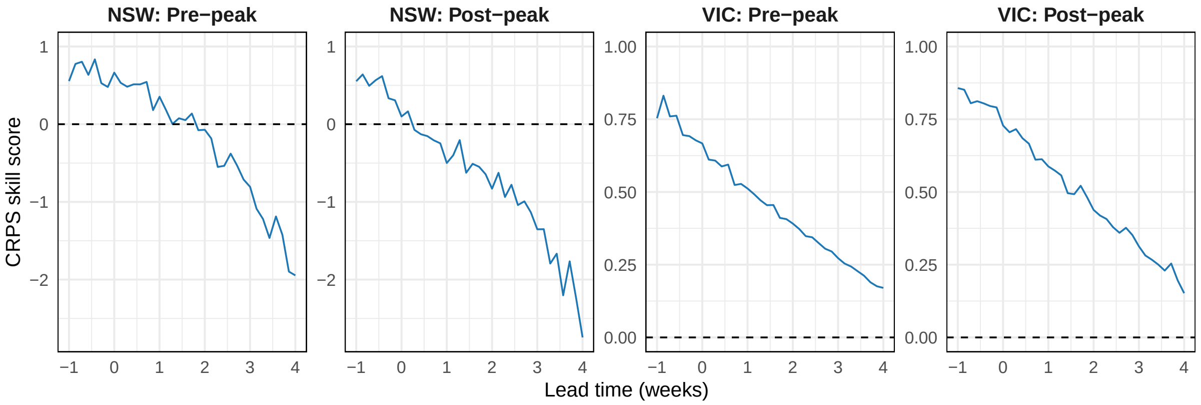

Figure S13: CRPS skill scores for the second wave in New South Wales and Victoria, shown seperately for data dates before and after the observed peak in each state.

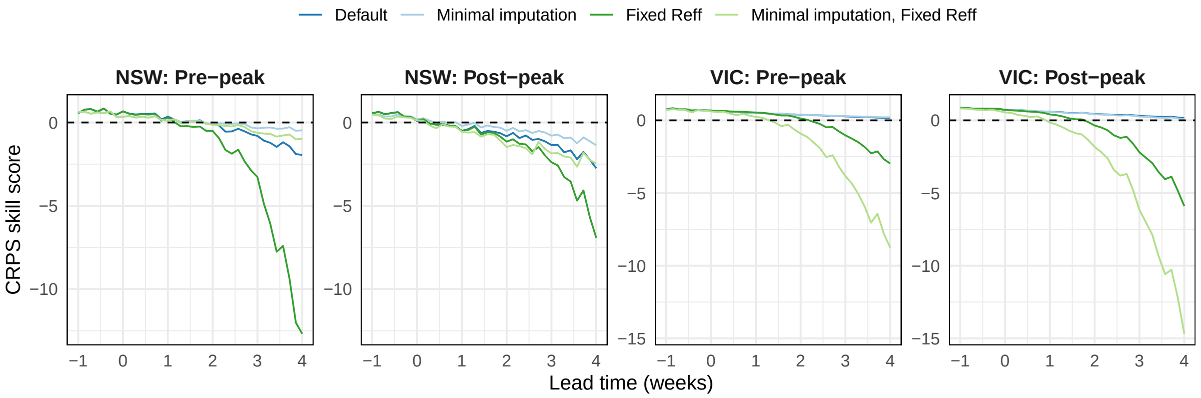

Figure S14: CRPS skill scores for the second wave in New South Wales and Victoria, shown seperately for each forecast model.

During the second wave (June to September, inclusive; 122 days) NSW reported 1–9 cases on 62 days (50.8%), and it was at these case levels that forecast skill was identified to be lowest across all jurisdictions (Figure S11). In contrast, Victoria reported 1–9 cases on only 14 days (11.5%), and reported at least 100 cases on 56 days (45.9%). Over this time period, the New South Wales forecasts out-performed the historical benchmark at shorter lead times (up to one week ahead), while in Victoria the forecasts consistently out-performed the historical benchmark at all lead times (Figure S13). By using our model to predict how transmission would change over the forecast horizon (relative to the current active-case R_{eff} estimate) forecast performance was greatly increased (Figure S14, compare blue lines to green lines).

S.3.5 Forecast skill by predicted local cases

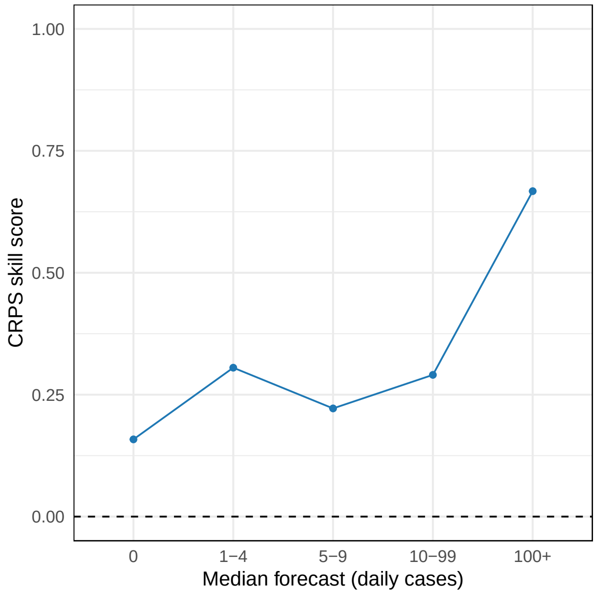

Figure S15: CRPS skill scores binned by the median forecast: 0 cases (81.2% of forecast days); 1–4 cases (6.9%); 5–9 cases (3.5%); 10–99 cases (5.5%); and 100+ cases (2.9%).

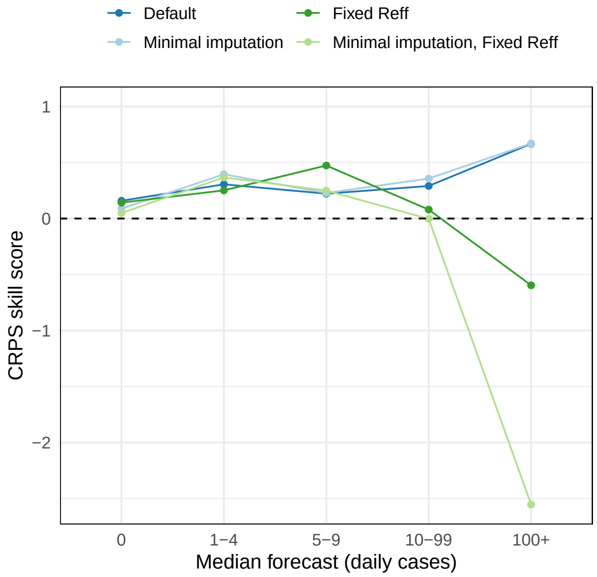

Figure S16: CRPS skill scores binned by the median forecast number of cases, shown separately for each forecast model.

When considering forecast skill as a function of the number of cases ultimately recorded for each day, as above, the results can only be evaluated in retrospect, since these numbers are unknown when the forecast is made. Accordingly, we also considered forecast skill as a function of the number of daily cases *predicted by the forecast model*, which may provide assistance when interpreting the forecast outputs in near-real-time. Our intent was to address the question "what does this forecast mean?" before the ground truth is known.

The study period comprised 26 data dates across 8 jurisdictions, and the forecast interval spanned 36 days, resulting in 7,488 predictions of daily case counts. We binned

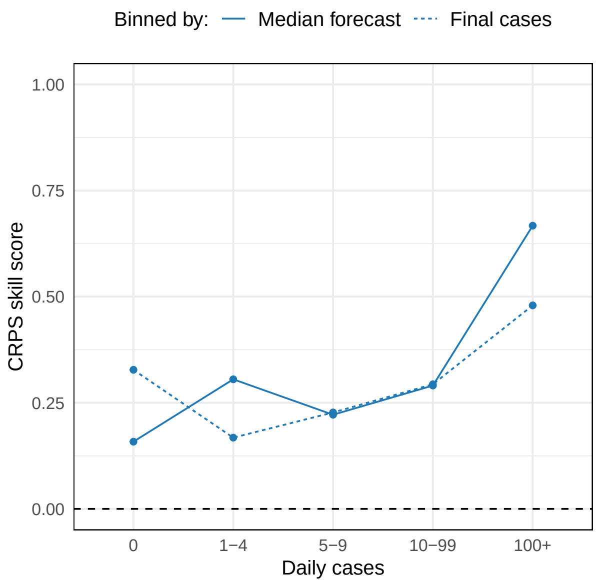

Figure S17: CRPS skill scores binned by the median forecast (solid line) and by the number of cases ultimately recorded (dashed line).

these forecasts by the median predictions, which was correlated with forecast sharpness (Figure S1) and was therefore also indicative of forecast uncertainty, and used the same bins as before: 0 cases; 1–4 cases; 5–9 cases; 10–99 cases; and 100+ cases.

As per the skill scores aggregated by the number of cases ultimately recorded for each day, the forecasts always out-performed the historical benchmark. The forecast skill was lowest when the median prediction was zero cases, and was highest when the median prediction was $\geq$ 100 cases, followed by 1–4 cases (Figure S15). Forecasts generated under the assumption that transmission would be sustained at the current active-case R_{eff} exhibited similar skill to the default model forecasts when the predicted case numbers were low, but their skill decreased as the predicted case numbers increased (Figure S16).

In comparison to the skill scores aggregated by the number of cases ultimately recorded for each day, the skill scores aggregated by the predicted case numbers exhibit similar trends for $\geq$ 5 cases (Figure S17). In comparison to a median prediction of zero cases, the skill score is higher when there are zero reported cases. And in comparison to a median prediction of 1–4 cases, the skill score is lower when there are 1–4 reported cases. This suggests that when given a near-real-time forecast, where the future case counts are truly unknown, we can plausibly estimate the likely forecast skill when there is a moderate degree of local transmission.

S.4 Effective reproduction number trajectories

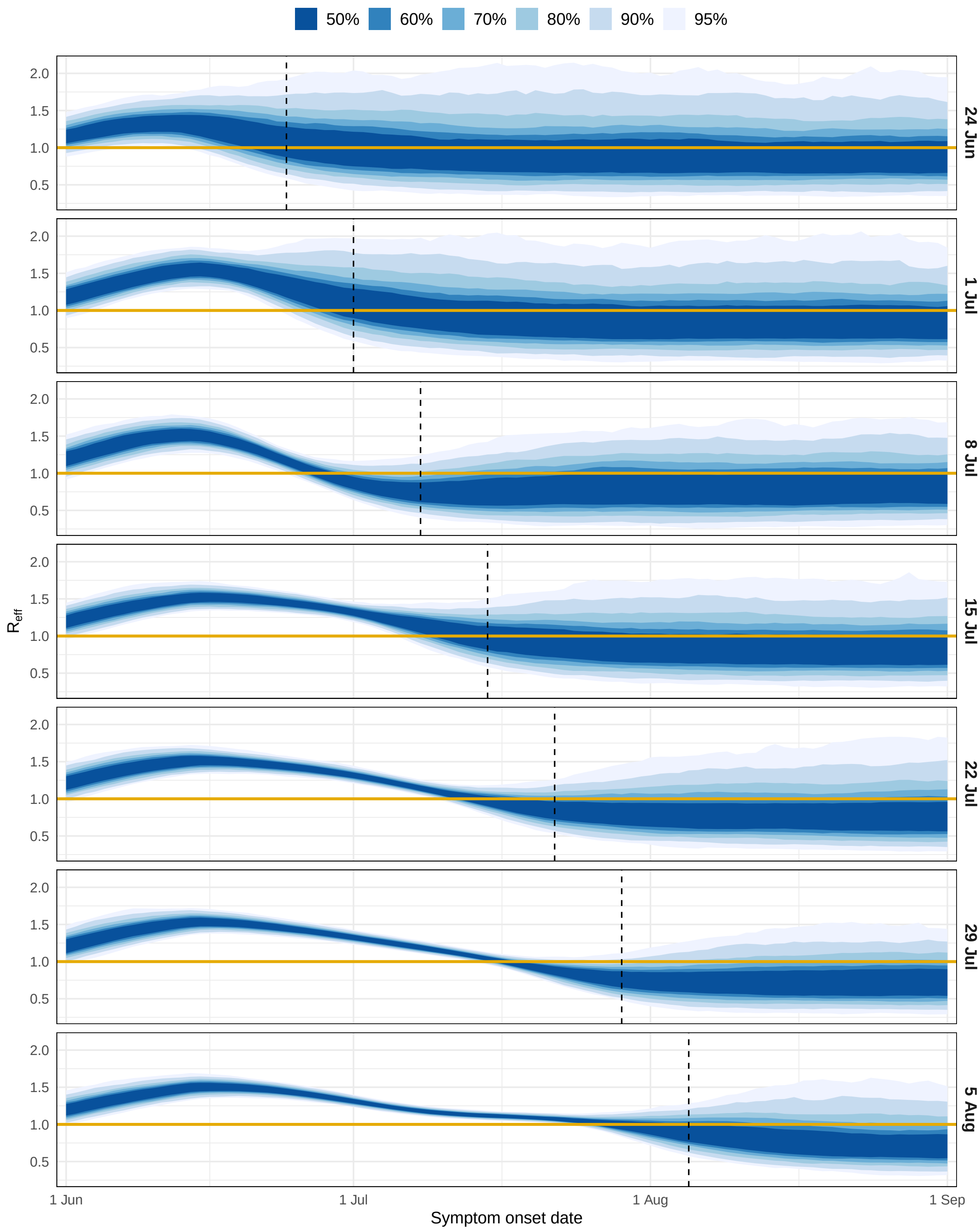

Figure S18: The R_{eff} trajectories for Victoria, shown separately for each data date (right-hand axis labels). Each set of trajectories is shown as a spread of quantiles (blue shaded regions). Vertical dashed lines indicate the data date, and horizontal dark yellow lines indicate $R_{\text{eff}} = 1$.

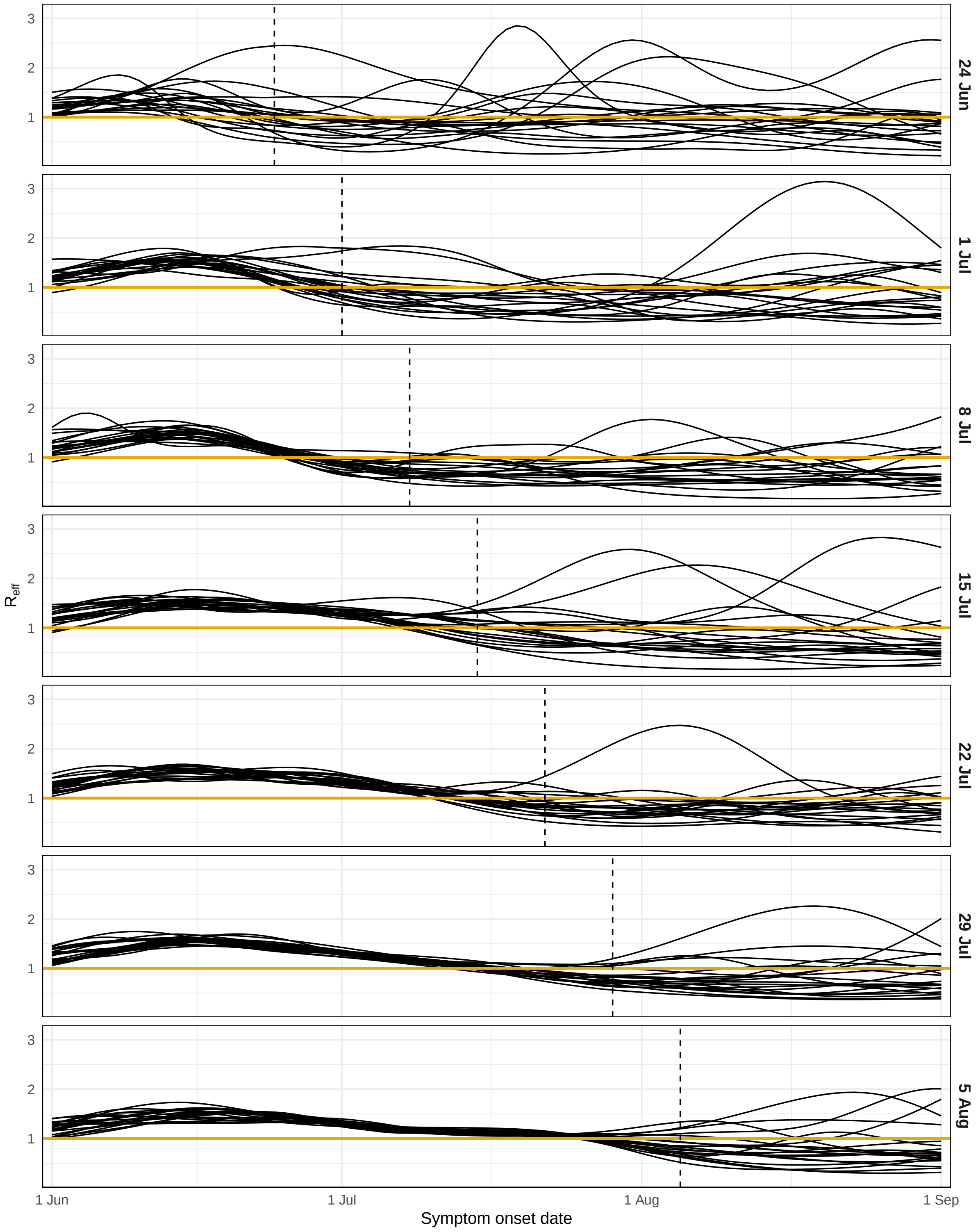

Figure S19: A sample of 20 R_{eff} trajectories for Victoria, shown separately for each data date (right-hand axis labels). Vertical dashed lines indicate the data date, and horizontal dark yellow lines indicate $R_{\text{eff}} = 1$.

S.5 Healthcare-seeking behaviour and COVID-19 tests

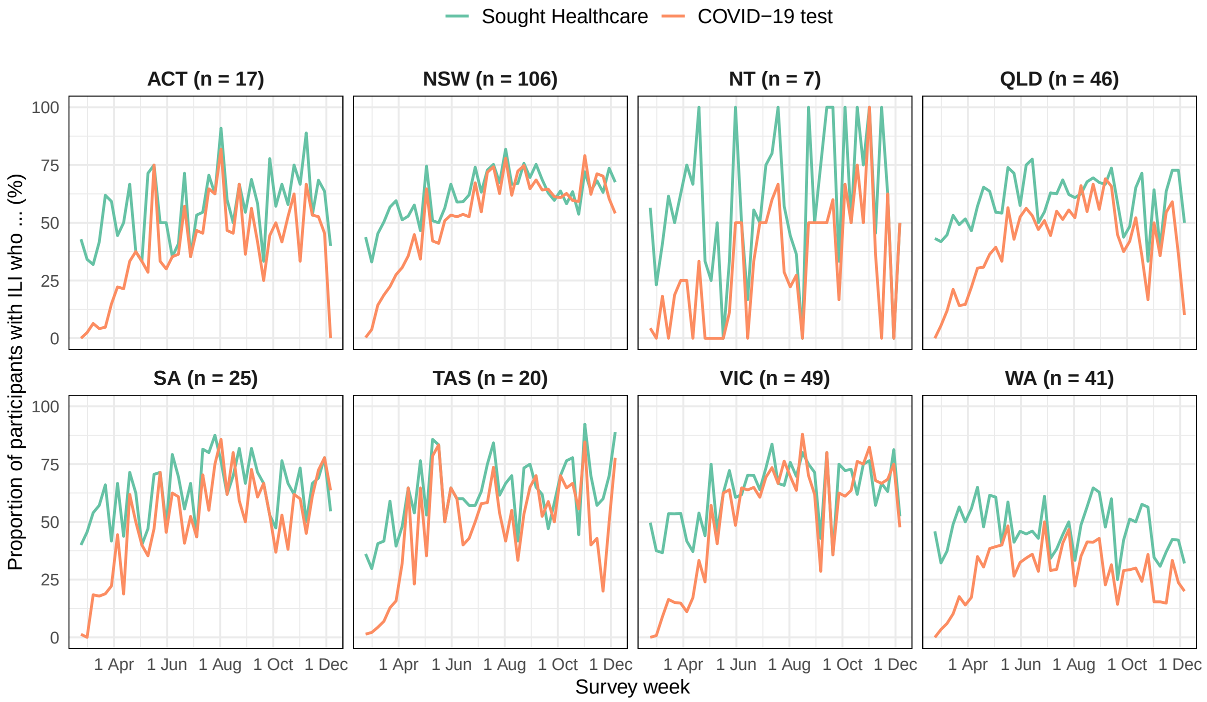

Figure S20: The proportion of Flutracking [2, 3] participants who reported ILI symptoms that (a) sought healthcare advice due to these symptoms (green lines); and (b) had a COVID-19 test (orange lines). The average number of participants n who reported ILI symptoms to Flutracking at each week is listed in the sub-plot captions. For comparison, in recent influenza seasons only 3–8% of participants who reported ILI symptoms had an influenza test [4].

References

1. Tong, H. *Non-linear time series: a dynamical system approach* ISBN: 9780198523000 (Oxford University Press, 1993).
2. Dalton, C. B., Carlson, S. J., Butler, M. T., Elvidge, E. & Durrheim, D. N. Building Influenza Surveillance Pyramids in Near Real Time, Australia. *Emerging Infectious Diseases* **19,** 1863–1865 (2013).
3. Dalton, C. B. *et al.* Insights From Flutracking: Thirteen Tips to Growing a Web-Based Participatory Surveillance System. *JMIR Public Health Surveillance* **3,** e48 (2017).
4. Moss, R., Zarebski, A. E., Carlson, S. J. & McCaw., J. M. Accounting for healthcare-seeking behaviours and testing practices in real-time influenza forecasts. *Tropical Medicine and Infectious Disease* **4,** 12 (2019).
